# Perspective: use of beef in a dietary intervention as an effective strategy for improving cognition in young adult females

**DOI:** 10.3389/fnut.2026.1770273

**Published:** 2026-04-08

**Authors:** Aaron Riviere, Steven E. Riechman

**Affiliations:** 1 Department of Nutrition, Texas A&M University, College Station, TX, United States; 2 Department of Health and Kinesiology, Texas A&M University, College Station, TX, United States

**Keywords:** beef, cognition, female, iron, visuospatial

## Abstract

Iron deficiency disproportionately affects young adult females and may impair cognitive performance. While supplementation has been studied, dietary interventions using iron-rich whole foods, particularly beef, remain underexplored. Beef provides highly bioavailable heme iron along with vitamin B12, zinc, choline, and creatine, nutrients implicated in neurotransmission, myelination, and cortical function. This review synthesizes evidence on beef consumption, iron status, and cognition that demonstrate improvements in attention, memory, and visuospatial processing in young adult females. We examine intervention doses, methodological differences, and limitations in current iron deficiency diagnostic thresholds. Despite mixed findings across studies, beef emerges as a feasible dietary strategy to support cognitive function in young adult females. Standardized cognitive assessments, longer interventions, and comparative analyses of alternative protein sources are needed to clarify the long-term cognitive benefits of beef. Considerations regarding red meat intake, sustainability, and evolving dietary patterns remain important. This review provides an evaluation of dietary iron from beef as a modifiable factor in cognitive performance. It also offers guidance for future research and raises concerns for balancing nutritional adequacy, health, and environmental considerations.

## 1 Introduction

Iron deficiency and anemia represent a major global health challenge, with a disproportionate burden among women of reproductive age. Globally, anemia impacts 24.3% of the global population, with prevalence among females aged 14–59 years (33.7%) substantially higher than among males of the same age (11.3%). The burden of anemia varies geographically, with the highest prevalence in Sub-Saharan Africa and South Asia, though clinically significant rates still occur in higher income countries (1). In the United States, anemia affects approximately 9% of the population, with a higher prevalence among females aged 12–19 (17.4%) and aged 20–59 (14.0%) than males of the same age groups (0.9 and 3.9% respectively). Despite global efforts to reduce anemia, prevalence among women of reproductive age has increased over time, rising from 7.8% in 2000 to 11.5% in 2018 (2).

Although anemia has multiple etiologies, iron deficiency is the leading cause worldwide, accounting for 66.2% of all anemia cases (1). Iron deficiency is also substantially more prevalent than anemia alone, impacting approximately 2 billion people globally and about 14% of the US population (3). In the United States, iron deficiency is particularly common among women of reproductive age, with a prevalence estimated at 34% compared to 3% among aged-matched males (4). Iron deficiency remains a persistent concern among women of reproductive age, spanning a continuum from depleted iron stores to overt anemia.

This deficiency has significant cognitive implications, as iron plays a key role in functions like oxygen transport, neurotransmitter synthesis, and myelination (5). Although iron supplementation is widely used to address deficiencies, iron-rich foods can offer not only highly bioavailable heme iron but also other nutrients that may support cognitive function (6, 7). Iron supplementation also has a greater risk for GI distress and iron toxicity, requiring individualized treatment based on physiological need and tolerance (8, 9). Iron supplementation risks as well as other barriers can lead to individuals stopping supplementation, with studies finding adherence ranging from 26 to >80% depending on the level of supervision of the researchers and education on the supplement (10).

Beyond iron status alone, higher risk for deficiencies in this population is especially concerning for brain health as there is also a sex gap in cognition function, with females tending to score lower in visuospatial cognitive tests (11, 12). Though the reason for the sex difference has not been fully explained, current evidence shows a multifactorial cause with biological and environmental origins (11). The difference has been linked to hormone levels, societal norms, and socioeconomic status, but nutrition’s role in this sex difference remains underexplored.

Much of the existing literature focuses on supplementation, leaving the question of whether dietary interventions with iron-rich foods can provide similar cognitive benefits. This paper aims to explore how beef, particularly its iron content, contributes to cognitive performance in young adult females.

## 2 Background: Iron in the brain and cognition

Iron is essential for brain function and cognitive performance due to its central role in cellular energy metabolism, structural development, and neurotransmitter synthesis. Through its oxygen carrying role in hemoglobin, iron supports aerobic metabolism required for numerous neuronal processes. At the cellular level, iron and oxygen availability directly influence mitochondrial function and energy production in neurons. In the hippocampus, iron deficiency has been shown to reduce mitochondrial size and mobility as well as disrupt mitochondrial fusion and fission. This leads to reduction in aerobic ATP production within dendrites and impairs dendritic growth and branching, processes critical for learning and memory (13).

Beyond neuronal metabolism, iron availability in the brain is regulated by glial cells, particularly astrocytes, which play a central role in maintaining cerebral homeostasis. Astrocytes function as key regulators of iron transport at the blood–brain barrier, producing ceruloplasmin, an enzyme that facilitates iron flux in and out of brain cells, and hepcidin, a hormone that reduces iron crossing the blood–brain barrier. Impaired astrocyte ceruloplasmin expression has been shown to reduce iron concentrations in the cerebral cortex and hippocampus, resulting in disrupted neurogenesis, impaired myelin development, and deficits in spatial learning and memory in adult mouse models (14). These findings highlight the importance of astrocyte mediated iron transport in supporting both brain structure and cognitive function.

Iron is also critical for oligodendrocytes, the glial cell responsible for myelination. Oligodendrocytes maintain large iron stores due to their functions as a cofactor for several enzymes involved in myelin synthesis and the high energy demands of myelin production. Iron deficiency during early development has been shown to impair oligodendrocyte maturation and reduce myelination, while iron deficiency in adulthood is associated with reduced remyelination. Together, these findings emphasize the importance of adequate iron for both neurodevelopment and maintenance of neural integrity across the lifespan (15).

In addition to its structural and metabolic roles, iron is required for dopamine synthesis through its involvement in iron-dependent enzymes. Reduced iron availability has been shown to decrease dopaminergic activity, particularly impacting the striatum, a region integral for complex learning and cognitive flexibility. Reductions in striatal dopamine function may then play a role in learning impairments seen in iron deficient populations (16). These mechanisms align with observational and intervention studies linking iron status to impairments in cognition. For instance, lower serum ferritin levels correlate with deficits in tasks requiring attention, memory, and spatial processing (6). Additionally, studies have shown that oral iron supplementation can improve cognitive outcomes such as executive function, attention, and memory (17, 18). While iron supplementation has demonstrated cognitive benefits, the extent to which iron rich whole foods, such as beef with its additional nutrients, can provide similar or additive effects remains insufficiently studied.

## 3 Nutritional role of beef in cognition

Beef provides highly bioavailable heme iron, which is more efficiently absorbed than the non-heme iron found in plant-based foods (7). Beyond iron, beef is rich in nutrients critical for cognitive health, such as vitamin B12, zinc, choline, and creatine (which is not the case for most plant-based protein sources). For young adult females, a 3-ounce cooked beef patty provides 100% of the recommended B12 intake, 66% of the recommended zinc intake, 16% of recommended choline intake, and 0.4 g of creatine, all of which play roles in brain function (18, 19). These nutrients have been shown to increase cognition in younger populations, especially when deficiencies are present (20, 21). In young adults, iron has been highlighted as a particularly concerning nutrient, with significant correlations between iron status markers in the blood and cognitive performance in young adults (22). There is minimal research in cognitive function in young adult females, especially focusing on whole food interventions. Given the known limitations of iron supplementation discussed earlier, whole-food interventions warrant further investigation. Food interventions are often through fortified foods including fortified lentils and salts, which can increase cognition in young adult females (23, 24). Only one previously published study has directly investigated beef as an intervention for cognition in young adult females. Blanton et al. provided beef to young adult females and measured motor skills, spatial planning, working memory, and sustained attention (6, 19). They found no consistent benefit of the beef intervention over the control arm, but there are a few limitations of the intervention. They only provided 3 oz. of beef three times a week and the control diet included other meat options, which also would have provided iron. They found that both groups had significant increase in cognition and that those with greater improvement in iron status had greater increase in cognition, regardless of the intervention. This study shows that multiple meat products may be beneficial for increasing cognition and iron status in this population (6, 19).

Several studies have looked at beef interventions in younger populations. A few studies in Kenyan school children found beef consumption improved cognitive outcomes, including memory, abstract reasoning, and academic performance (7, 20, 25). Two of these studies in Kenyan school children provided 2 oz. of beef five times a week (7, 25) while the other study provided a meat mixture in the form of a traditional dish (20).

A systematic review of red meat and cognition also noted inconsistencies in adult populations, underscoring the need for more robust intervention designs across all life stages (26). The systematic review pointed out various methodological differences in cognitive tests, dose of beef, control food, and dietary control. They also reported strong bias in the previous studies. Many of the intervention studies scored moderate to high in risk of bias from the randomization process, deviation from intended intervention, bias on measurement of outcomes, and bias due to selective reporting. Observational studies scored moderate to high in risk of bias from bias due to confounding, bias in selection of participants, bias in classification of exposures, and bias in reported results. Overall, the systematic review called for better controlled studies with consistent cognitive measures (26).

A recent pilot study by our group investigated the effects of adding a daily 3-ounce cooked beef patty to the habitual diets of healthy young adult females for 30 days. Visuospatial cognitive performance was assessed using the Neurotracker™ system, a 3D multiple objects tracking test (27). Neurotracker™ measures the ability of a participant to track the spatial location of four moving target spheres while four same colored distractor spheres interact with the targets at a given speed within a 3D virtual cube. The spheres pass in front of or behind each other as well as collide with each other and the edges of the screen. After 6 s of movement, the participant must select the four target spheres. If the subject selected the correct spheres, the speed of sphere movement increased for the next trial. If one or more spheres was incorrectly chosen, the speed of sphere movement decreases for the next trial based on an adaptive staircase protocol (28). The beef-consuming group demonstrated significant improvements in visuospatial cognition compared to a control group consuming a macronutrient equivalent soy-based vegetable patty. The beef group had a higher average Neurotracker score, 1.55 ± 0.29 vs. 1.38 ± 0.29 *p* = 0.008, higher maximal Neurotracker score, 2.01 ± 0.35 vs. 1.82 ± 0.42 *p* = 0.032, higher score of the last 3 Neurotracker sessions, 1.75 ± 0.31 vs. 1.43 ± 0.31 *p* < 0.001, and greater improvement from across the intervention, 0.45 ± 0.32 vs. 0.21 ± 0.19 *p* < 0.001. Interestingly, these cognitive benefits occurred despite no significant changes in blood iron levels in a subset of the beef group, suggesting that other nutrients in beef may have also contributed to the observed effects or that the subset was too small to find significant differences in blood values (unpublished). Additionally, the females consuming beef patties performed closer to males in past studies using Neurotracker, with males having an average score of 1.77 ± 0.34, a maximal score of 2.42 ± 0.44, a final score of 1.89 ± 0.36, and a change in score of 0.26 ± 0.30. The increase in female Neurotracker scores shows a potential nutritional intervention to narrow the cognitive sex gap previously observed in visuospatial domains (unpublished).

Compared to plant-based protein sources, beef’s nutrient profile offers distinct advantages. A soy-based vegetable patty, for instance, lacks creatine and provides significantly lower amounts of B12 and zinc. Beef also has been described as having a complex food matrix that allows for benefits beyond single nutrients such as the nutrient density of beef to provide bioavailable micronutrients that are commonly deficient in different populations (29). Other animal sources can serve to increase cognition as well. A study supporting the benefit of red meat added to a healthy diet provided a Mediterranean diet plan with 2–3 servings of lean pork a week in add in a population of adults aged 45–80 years old. The Mediterranean and pork diet was compared to a low-fat diet based on the PREDIMED studies. The research group found that the Mediterranean diet plus the pork improved cognitive processing speed and better emotional functioning (30).

## 4 Challenges and limitations

### 4.1 Research limitations

While there is growing evidence that iron and beef can play a role in supporting cognition in young adult females, several limitations in the research persist. First, many studies rely on short-term interventions, often lasting only a few weeks or months. These limited durations may fail to capture long-term cognitive benefits or changes in biomarkers such as ferritin levels, which can take months to stabilize. Additionally, the red blood cell lifecycle (~120 days) suggests that longer studies are needed to observe the full physiological impact of iron and beef consumption.

Second, there is significant variability in how iron deficiency is defined across studies and disagreement on clinically relevant iron levels, with diagnostic thresholds for ferritin levels ranging from 15 to 50 μg/L. While ferritin levels below 30 μg/L are commonly used, some researchers suggest raising this threshold to better capture cases of subclinical deficiency (31, 32). Evidence also indicates that iron absorption increases when ferritin levels fall below 50 μg/L, suggesting that even small reductions in iron stores can trigger compensatory mechanisms (32). This highlights the need for further investigation into the cognitive effects of subclinical iron levels. This discrepancy also makes it complicated to compare results across studies and may lead to underestimation or overestimation of the prevalence of iron deficiency in study populations.

Finally, many studies use cognitive assessments that differ widely in their sensitivity and scope. For example, the Stroop Color and Word test or Raven’s progressive Matrices and Vocabulary tests only target certain cognitive domains, inhibiting cognitive interference and “fluid intelligence” (33, 34). These more common, simpler tests with outputs are not able to measure complex cognitive processes that more complex assessments can quantify. Neurotracker, used in the previously mentioned study, measures the participant’s ability to maintain attention on multiple objects while ignoring distractors. The participant must also maintain attention in a three-dimensional space which makes it a more applicable test to real-life cognitive needs. Neurotracker has been correlated with performance in brain injury recovery (35), driving (28), sports performance (36), and learning (37).

### 4.2 Broader challenges

There are several counterarguments against promoting beef consumption as a primary source of iron for cognitive health. One common concern is the potential health risks associated with red meat intake, including associations with cardiovascular disease and cancer. While some epidemiological studies suggest a link between high red meat consumption and negative health outcomes, these findings often fail to account for confounding dietary factors, such as processed versus unprocessed red meat and overall dietary patterns or other unhealthy diet and lifestyle factors that contribute to the negative health outcomes (38). A systematic review of the long-term intake of red meat and risk of dementia found that a higher chronic intake of red meat increased the risk of dementia and cognitive decline, but that processed red meat had a much stronger association when subtypes of red meat were analyzed. The authors described likely mechanisms causing the decline, such as higher saturated fat, sodium, and nitrates, which all are in greater quantity in processed meat than in lean beef (39). Further research on the leanness and processing of red meat may drive the associations with cardiovascular disease and mortality. Researchers investigating the impact of leanness of red meat found that when individuals removed visible fat from red meat, there was no longer a significant association with cardiometabolic outcomes (40). Unprocessed red meat has also been found to have no association with mortality and major CVD outcomes, though processed red meat still was positively associated (41). Additionally, reviews have investigated the impact of red meat on risk for cardiovascular disease, cancer, and all-cause mortality. The authors found that based on current evidence, unprocessed red meat intake has a very small effect size on each of the outcomes with low to very low certainty of evidence (42, 43). While there is still a need for further research investigating red meat as part of the whole diet, some research is already showing diet quality plays a significant role on the impact of red meat. A study investigating adding green leafy vegetables to a diet high in red meat could reduce oxidative damage to DNA and reduce markers of inflammation (44). Red meat as part of a diet high on the Healthy Eating Index was found to have no negative effects on BMI or gut microbiome and also have higher levels of select nutrients when compared to the same diet quality without red meat intake (45). These studies support red meat as part of a healthy diet that follows recommendations for variety, balance, and moderation.

Another counterpoint is that iron can be obtained from alternative sources, including plant-based foods and supplements. While plant sources such as lentils, spinach, and fortified cereals contain non-heme iron, it is less bioavailable than heme iron from animal sources. The presence of phytates and polyphenols in plant foods can further inhibit iron absorption, making it challenging for some individuals, particularly young menstruating adult females who have higher iron needs, to maintain optimal iron levels through plant-based diets alone (46). Previous research has shown that a vegetarian diet with equivalent iron consumption to an omnivore diet leads to significantly less serum ferritin levels (47). Unlike plant sources, iron supplementation can effectively increase iron related blood values quickly and is the recommended intervention for clinical conditions. Even with iron supplementation, dietary changes would still be recommended to support iron absorption and improve intakes for after treatment (48). This review focused on subclinical blood values in a population with frequent inadequate iron intake who is at a greater risk for developing iron deficiency. Iron supplementation widely distributed to all young adult females would not fit current supplementation guidelines and would increase the risk for GI distress and iron toxicity (3). Improving dietary iron intakes would serve to help reduce the risk of iron deficiency and also be part of treatment in a clinically deficient individual.

Sustainability and environmental concerns also play a role in the debate surrounding beef consumption. Beef production has been linked to higher greenhouse gas emissions compared to plant-based proteins (49). However, emerging research suggests that sustainable livestock management practices can mitigate some of these environmental impacts (50). Additionally, the nutrient density of beef means that smaller portions may provide the necessary iron and cognitive benefits without excessive caloric consumption, especially when compared to plant products. Using NHANES data from 2001–2018, Agarwal and Fulgoni found females older than 18 consumed 18% of their B12, 6.4% of their iron, 12% of their protein, 4.6% of their energy, 19% of their zinc, 8.9% of their saturated fat, and 2.3% of sodium from consuming beef products. Processed beef in this study provided more saturated fat and sodium than other foods investigated. Unprocessed beef did not provide any more saturated fat or sodium per 100 kcal than other food options but did provide more protein and other key nutrients (51). Cost and accessibility may also influence the feasibility of increasing beef consumption in some populations. This should be considered in future strategies but a recent project in low income countries showed that improving combined animal and vegetable gardening can positively impact both food security and health status, including anemia (52).

Addressing these counterarguments requires a nuanced discussion that considers both the potential benefits and drawbacks of beef consumption. Another challenge is the growing decrease in beef consumption in multiple groups, including adult females. According to NHANES data, beef intake decreased by 5.7 g/day from 2001 to 2018 in adults aged 18–59 years with males on average consuming 60.8 ± 1.7 g and females consuming significantly less at 33.1 ± 1.1 g per day (53). Addressing the above concerns and creating better public education around the actual benefit and risk is important to address potential nutrient inadequacies that can influence cognition. A balanced approach that emphasizes dietary diversity, including both animal and plant-based iron sources, may be the most effective strategy for optimizing cognitive health in young adult females.

## 5 Future directions

Future research should explore the long-term effects of beef consumption on cognition, particularly in populations with varying baseline iron levels. Comparative studies examining different protein sources, such as chicken or pork, could help isolate the specific contributions of beef’s nutrient matrix to cognitive health. Mechanistic studies investigating the roles of creatine, B12, and other nutrients in beef could further elucidate the pathways through which it supports cognition. There is emerging evidence of creatine’s role in brain health, but mostly in older adults (54). Future studies should also examine changes in biomarkers of iron status (such as ferritin and hemoglobin) alongside cognitive outcomes and monitor lipid biomarkers and other indicators of cardiometabolic risk. Future interventions should therefore evaluate both cognitive outcomes and broader cardiometabolic health indicators to ensure that potential benefits do not come at the expense of overall health.

From a practical standpoint, dietitians should consider dietary recommendations that incorporate iron-rich whole foods like lean beef, particularly for young adult menstruating females at greater risk for deficiency. Education on the bioavailability of heme iron, how to choose lean and unprocessed sources of heme iron, and strategies for optimizing iron absorption (e.g., pairing with vitamin C sources) should be integrated into dietary counseling.

Policymakers and public health officials should support nutritional guidelines that reflect the cognitive benefits of iron-rich foods and work to ensure that individuals at risk of iron deficiency have access to diverse and nutrient-dense protein sources. Public health initiatives could include campaigns on iron deficiency awareness and dietary strategies to mitigate its effects.

Researchers should focus on developing sensitive and standardized cognitive assessment protocols to ensure consistency across studies examining diet and cognition. Additionally, long-term dietary intervention studies should be prioritized to better capture the sustained cognitive effects of beef consumption. Extending interventions to include males and other demographic groups could provide a broader understanding of beef’s cognitive benefits. Further research into the sustainability of beef production and its role in balanced dietary patterns could address public concerns and support its inclusion in evidence-based dietary guidelines (38, 49).

## 6 Conclusion

Beef offers a nutrient-dense food matrix that supports cognitive performance and may be particularly important for reproductive-age females at risk for iron deficiency. Evidence from recent research demonstrates significant improvements in visuospatial cognition following beef consumption, likely due to the synergistic effects of its nutrients. Addressing dietary gaps in iron and related micronutrients through whole-food solutions like beef has the potential to improve cognitive health and quality of life.

However, broader food trends, such as the increasing shift toward plant-based diets, raise important considerations. While plant-based diets offer many health and environmental benefits, they often provide less bioavailable iron, which may increase the risk of iron deficiency and its associated cognitive impacts, particularly for young adult females. Future dietary recommendations should balance the benefits of plant-based eating with strategies to ensure adequate iron intake, whether through fortified foods, supplementation, or strategic inclusion of bioavailable iron sources like beef.

Further research is needed to optimize intervention strategies and address broader societal concerns surrounding red meat consumption, ensuring that dietary recommendations reflect both nutritional needs and evolving food preferences.
